# Microbial communities evolve faster in extreme environments

**DOI:** 10.1038/srep06205

**Published:** 2014-08-27

**Authors:** Sheng-Jin Li, Zheng-Shuang Hua, Li-Nan Huang, Jie Li, Su-Hua Shi, Lin-Xing Chen, Jia-Liang Kuang, Jun Liu, Min Hu, Wen-Sheng Shu

**Affiliations:** 1 State Key Laboratory of Biocontrol, Key Laboratory of Biodiversity Dynamics and Conservation of Guangdong Higher Education Institutes, College of Ecology and Evolution, Sun Yat-sen University, Guangzhou 510275, People's Republic of China; 2 These authors contributed equally to this work.

## Abstract

Evolutionary analysis of microbes at the community level represents a new research avenue linking ecological patterns to evolutionary processes, but remains insufficiently studied. Here we report a relative evolutionary rates (rERs) analysis of microbial communities from six diverse natural environments based on 40 metagenomic samples. We show that the rERs of microbial communities are mainly shaped by environmental conditions, and the microbes inhabiting extreme habitats (acid mine drainage, saline lake and hot spring) evolve faster than those populating benign environments (surface ocean, fresh water and soil). These findings were supported by the observation of more relaxed purifying selection and potentially frequent horizontal gene transfers in communities from extreme habitats. The mechanism of high rERs was proposed as high mutation rates imposed by stressful conditions during the evolutionary processes. This study brings us one stage closer to an understanding of the evolutionary mechanisms underlying the adaptation of microbes to extreme environments.

Understanding the mechanisms underlying the adaptation of microbes to extreme environments is of fundamental importance from both evolutionary and ecological perspectives^1^,^2^. Despite the philosophical controversy over the definitions of “extreme”, a physical definition of “extreme” as unfavorable environmental factors that depress the ability of organisms to function is commonly used in ecological studies^3^. Several typical environments, including saline lake, acid mine drainage (AMD) and hot spring, are widely perceived as extreme environments for their stressful factors such as extensive osmotic stress, low pH and high temperature, respectively^3^,^4^,^5^. Over the past decade, an increasing number of studies have been focused on how microorganisms populating extreme environments cope with stress^6^. Several works have found that genome plasticity, including codon bias, nucleotide skew and horizontal gene transfers (HGTs), enables evolutionary adaptation to extreme conditions^7^,^8^. A more recent study highlighted the role of frequent recombination in rapid adaptation within AMD communities since the bacterial hybrids showed remarkable ecological success^9^. However, general patterns have not been detected regarding the adaptive mechanisms of microbes living under the harsh conditions. This is likely due to the variety of selective pressures in extreme environments. For most microbes, adaptation to such stressful environments is a highly dynamic and complex process that involves the interaction of multiple evolutionary forces^10^,^11^.

In contrast to the examination of the adaptive mechanisms of specific taxa individually, the study of microbial evolution at the community level represents a new research approach that links ecological patterns to evolutionary processes^2^. Indeed, prokaryotes typically evolve as consortia comprising a phylogenetic mosaic in natural environments^12^. These heterogeneous groups have been described as the units responsible for habitat selection^13^ and thus are likely to represent the true units of evolution^14^. Therefore, metagenomics approaches that involve sampling the genetic content of the whole community inhabiting natural environments have potentials in shedding light on the integrative aspect of microbial evolution.

Although comparative metagenomics analyses are providing valuable insight into the adaptive strategies of microbes in their natural settings^8^,^15^,^16^, the question of how environments may impact the evolution of microbial communities remains unanswered. The exploration of adaptive fingerprints in natural communities has been hindered by the fact that rapidly evolving genetic modules are difficult to capture^17^. Additionally, direct measurement of the absolute rates of molecular evolution in natural assemblages is plagued by the problem of complex phylogenetic composition and the necessity of long-term tracking^9^. In contrast, relative evolutionary rate (rER) has been shown to enable a robust assessment of evolutionary differences among lineages^18^. A previous study of community rERs through a comparison of the branch length of phylogenetic marker genes^13^ indicated that microbes from the ocean surface evolve faster than those from other habitats, including AMD environment. However, a sampling bias may have arisen due to the overrepresentation of pathogen genomes in the reference tree, making the previous results questionable.

To date, few studies have attempted a direct comparison of microbes from extreme conditions with their counterparts in relatively benign environments to explore microbial adaptation and evolution at the community level^19^. Furthermore, the relatedness between environment and evolution tempo remains poorly understood. The increasing amounts of metagenomics and fully sequenced genome data now allow us to systematically explore these important but unsolved questions. This study has illustrated the differences in rERs between microbial communities from extreme and normal environments based on an in-depth comparative analysis of 40 metagenomic samples from multiple heterogeneous habitats. The rERs assessment that we have outlined here is a necessary step toward a comprehensive understanding of the mechanisms of evolutionary change that underlie the adaptation of microbes to extreme conditions.

## Results

### Habitat profiling and evolutionary characterization of natural microbial communities

**Figure 1:**
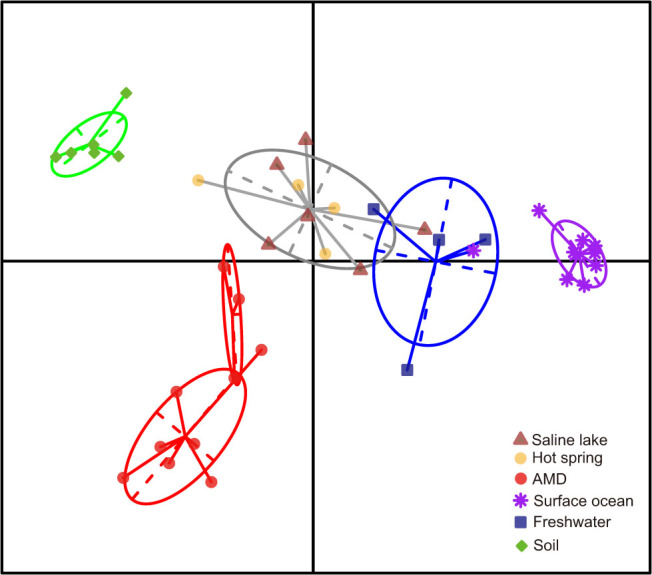
Discriminant analysis of principal components (DAPC) based on relative abundances of COG categories showing habitat profiling of 40 microbial communities across six habitats. The six habitats were denoted by corresponding cluster and colors.

**Figure 2:**
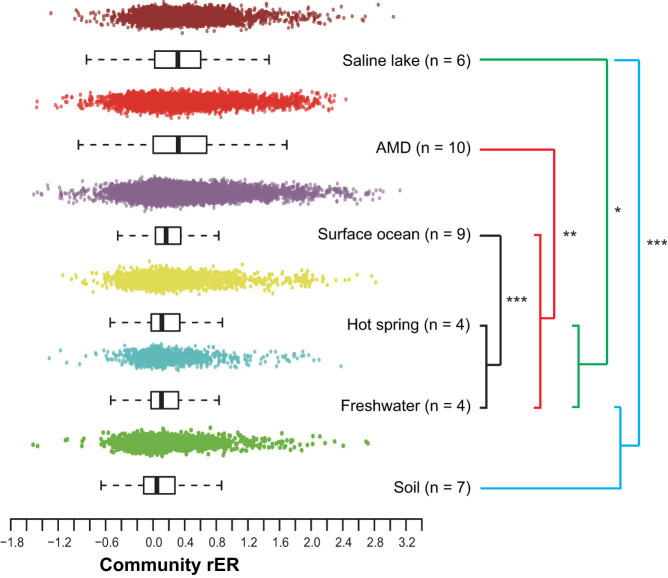
Scatter plot showing the distribution of rERs of the six habitat categories, based on the pooled data of all samples in each category. The 5%, 25%, 50%, 75%, and 90% quartiles are indicated. The significant differences of rERs among different habitat categories were determined using pairwise Mann–Whitney *U*-tests based on the average rER for each habitat as displayed in Supplementary Table S1. (*P < 0.05; **P < 0.01; α = 0.05, two-tailed. All *P*-values were adjusted for multiple testing using the “BH” correction in R. Detailed *P*-values were listed as follows: saline lake vs. freshwater, 0.029; saline lake vs. soil, 0.010; saline lake vs. hot spring, 0.033; AMD vs. hot spring, 0.007; AMD vs. surface ocean, 0.020; AMD vs. freshwater, 0.007; AMD vs. soil, 0.007; hot spring vs. surface ocean, 0.028; hot spring vs. soil, 0.040; surface water vs. freshwater, 0.017; surface water vs. soil, 0.007; freshwater vs. soil, 0.020).

The 40 communities were clustered based on the functional distance matrix of the COG categories to provide a habitat profile. The exploratory clustering pattern generally matched the corresponding six habitats: Saline lake, AMD, surface ocean, hot spring, freshwater and soil (Figure 1). For an overall assessment of the evolutionary pattern of these natural communities, we estimated the community-scale rER, dN/dS, HGTs (indicated by the occurrence of transposases encoding genes) and species diversity (estimated via ACE) (See Methods section for details). Results showed that microbial communities from different habitats exhibited distinct evolutionary variations, ranging from evolutionary tempo to species diversity (Supplementary Table S1). Firstly, the rER measures the evolutionary tempo of organisms in natural communities based on the estimation of accumulated number of sequence changes in a phylogenic reference tree. Our analysis revealed different evolutionary rates for microbes dwelling in different habitats. In particular, organisms populating AMD generally evolve faster than those from other habitats except saline lake (pairwise Mann–Whitney *U*-tests, *P* < 0.05 after correction for multiple testing, α = 0.05, one-tailed) (Figure 2). In contrast, the seven soil communities (including farmland, forest and grassland) displayed fairly stable rERs that were lower than those of aquatic environments (pairwise Mann–Whitney *U*-tests, *P* < 0.05 after correction for multiple testing, α = 0.05, one-tailed) (Figure 2). Secondly, metagenome-scale pairwise dN/dS analysis showed an overwhelming purifying selection in these communities, suggesting that the purging of deleterious mutations plays a key role in community evolution. Thirdly, the estimated transposases levels differed remarkably among the six habitats, with a range between 1.0% in AMD and 0.06% in surface ocean. These values were comparable to those previously reported for similar environments^15^,^20^. The distinct transposases levels might reflect that HGTs were ecologically structured. Similarly, the difference in species diversity might be attributable to the distinct environmental conditions associated with the diverse habitats. Overall, our metagenome-based characterization of natural communities provided an initial look at the evolutionary patterns of organisms living in different environments.

### Environmentally dependent rERs of microbial communities

**Figure 3:**
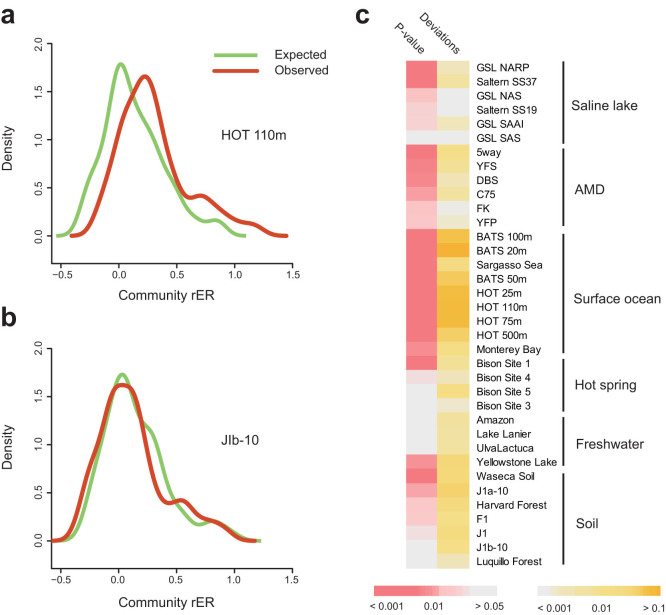
The rERs of natural communities apparently deviating from the expected values of the simulated samples. Of all 36 samples (the five subsamples from AMD C75 were pooled), 28 (78%) deviated from expectations (two-sided Kolmogorov-Smirnov tests, *P* < 0.05, α = 0.05). (a) HOT 110 m is shown as representative of the deviated groups, and (b) soil J1b-10 represents those that are consistent with expectations. (c) The detailed *P*-values and deviations (denoted by median) are illustrated in the heatmap.

The rER analyses revealed a signal of generally similar community rERs within the same habitat category (Supplementary Table S1), except that two of the saline lake samples exhibited inconsistent rates. The saline lake communities were sampled across a considerably wide range of salinity gradient and harbored large variance inherently^4^. Thus, the inconsistent rERs in the saline lake habitat appeared to reflect the impact of heterogeneous environmental conditions on genome evolution. In addition, communities from different habitats displayed distinct rERs (Figure 2). Further analyses using Spearman rank correlations showed a significant relatedness between habitats and community rERs (permutation test, *R* = 0.49, *P* < 2.2E-16, α = 0.001). To test whether the heterogeneous phylogenetic compositions of the various communities have a major influence on the above trends, we subsequently estimated the expected distributions of rERs for all samples by simulating the communities from weighted datasets and the matching corresponding phylogenetic compositions (see Methods section). Of all 36 samples (the five subsamples from AMD C75 site were pooled due to the small numbers of marker gene fragments), 28 (78%) deviated significantly from expectations (pairwise Kolmogorov-Smirnov tests, *P* < 0.05 after correction for multiple testing, α = 0.05, two-tailed) (Figure 3), suggesting that the pattern of rERs cannot be well explained by the distinct phylogenetic structures of the communities. These results indicated that the *in situ* rERs of microbial communities were largely environment dependent.

### Higher rates of evolution in extreme habitats than in normal habitats

**Figure 4:**
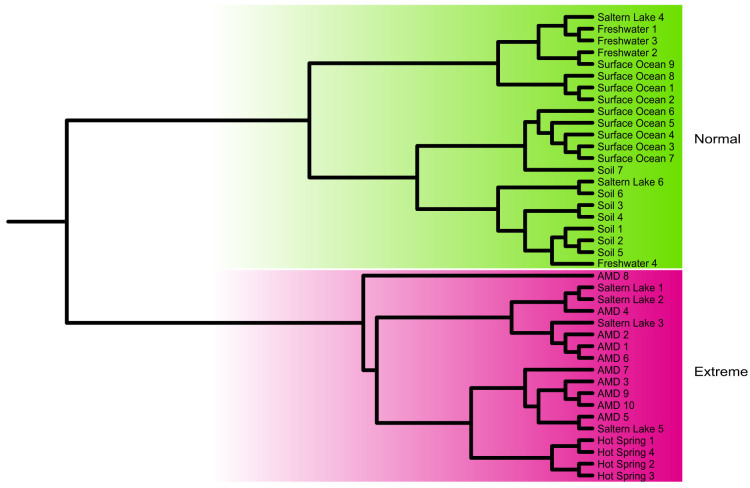
Clustering analyses based on the community-scale evolutionary variables (rER, dN/dS, transposases, and species diversity) generally divide the samples into the categories of “extreme” (AMD, hot spring, saline lake) and “normal” (surface ocean, freshwater, soil).

**Figure 5:**
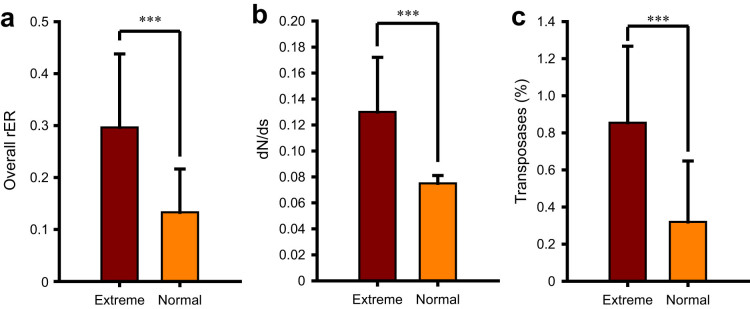
Detection of significantly higher overall community rER, dN/dS and relative abundance of transposases in extreme habitats than in normal ones (Mann-Whitney *U*-tests; ***P < 0.001. *P*-values were equal to 2.81E-04, 2.77E-05, 2.623E-05 respectively. α = 0.001, one-tailed).

An exploratory clustering analysis based on the four community-scale evolutionary variables (Supplementary Table S1) showed that the 40 samples were generally clustered into two groups (Figure 4), implying two different evolutionary patterns for these microbial communities. One group encompassed the samples from extreme habitats (saline lake, AMD and hot spring), and the other group included the samples representing relatively benign environments (surface ocean, freshwater and soil) (Figure 4). Quantitative comparison of the evolutionary differences between the two groups further revealed that microbes living in the extreme and normal habitats had an average rER of 0.296 and 0.133, respectively, indicating that organisms thriving under the harsh conditions evolve significantly faster (Mann–Whitney *U*-tests, *P* = 2.81E-04, α = 0.001, one-tailed; Figure 5a). Additionally, significantly higher dN/dS and transposases level were observed in the extreme habitats (Mann–Whitney *U*-tests, *P* = 2.77E-05 for dN/dS; *P* = 2.623E-05 for transposases level, α = 0.001, one-tailed; Figure 5b, c), reflecting more relaxed purifying selection and frequent HGTs in these extraordinary environments.

**Figure 6:**
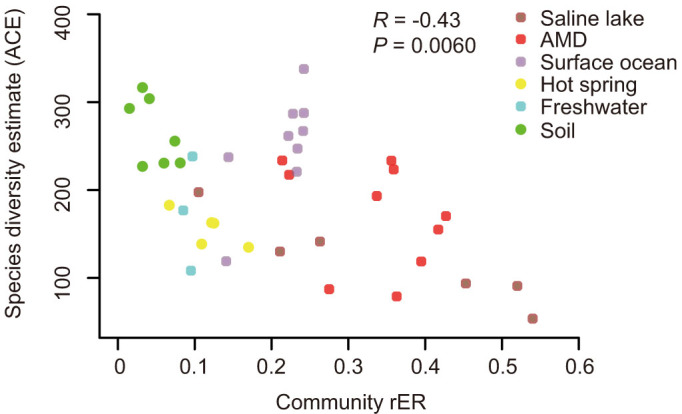
Correlation between community rERs of the six habitat categories and their species diversity estimates (ACE).

Our analysis also revealed interesting negative correlations between species diversity (ACE index) and community rERs (*R* = −0.43, *P* = 6.00E-03, α = 0.001; Figure 6), which implies that the evolutionary tempo in low diversity microbial communities was generally higher than that in more complex communities. This observation coincided with our expectation since habitat conditions could be generally reflected by community complexity in this study. This was supported by the finding that extreme environments exhibited generally lower diversity compared to normal environments (ACE index 152 vs. 240, Mann-Whitney *U*-tests, *P* = 1.449E-05, α = 0.001, one-tailed) (Supplementary Table S1).

### Case study of AMD communities

**Figure 7:**
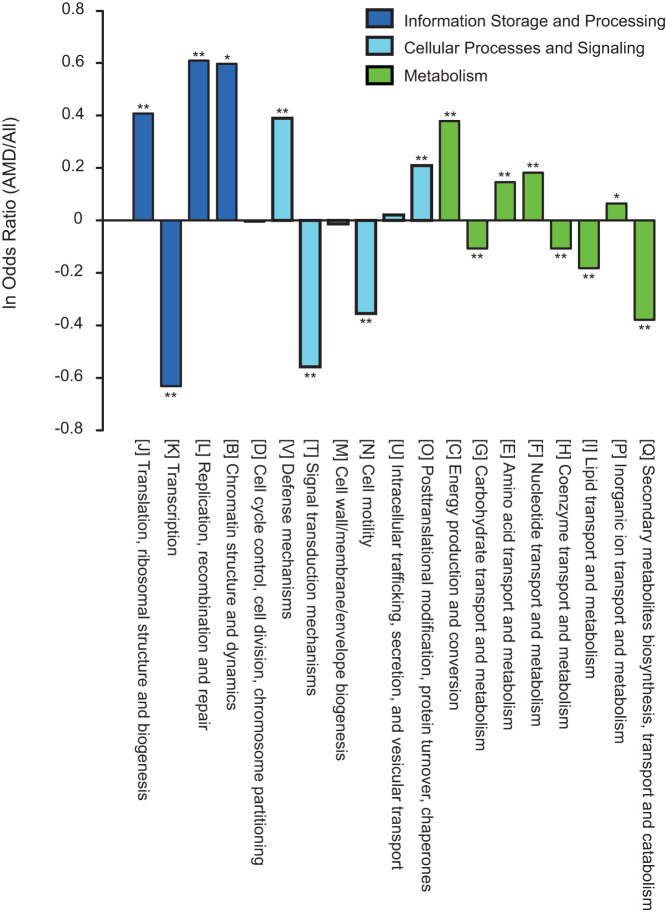
Odds ratio of the AMD genes compared to those from all sequenced prokaryotes for the genes annotated as COG functional categories. Asterisks denote a significant deviation from the null hypothesis (ln odds ratio = 0) (one-tailed Fisher exact test; *P < 0.01; **P < 0.001, α = 0.01).

The AMD communities were found to be highly enriched in genes for replication, recombination and repair compared to all sequenced prokaryotes (Figure 7), reflecting the necessity of evolving extensive DNA repair systems to cope with the harsh conditions. Similarly, the overrepresentation of genes that code for post-translational modification and molecular chaperones likely arose to redress incorrect protein folding partly due to the oxidative stress in the AMD environment. In contrast, genes related to transcription, signal transduction, secondary structure and related processes were found significantly underrepresented in the AMD communities (Figure 7). Differential gene loss and overlapping genes in AMD habitats could likely be means of directionally retaining indispensable genes and compressing accessory genetic information as the result of habitat selection and evolutionary pressure to minimize genome size^21^, suggesting that adaptive specialization in metabolism is important to adaptation to stressful environments.

## Discussion

Our community-scale analyses have revealed the overall rERs of natural microbial assemblages and their relatedness with diverse environments. It should be noted, however, that the calculation of rER of each phylogenetic marker gene sequence is dependent on the differences of branch length comparing to the relatives in the reference tree. Consequently, the rERs assessment may be biased due to the poor representation of organisms from relevant environments in the reference tree and the imprecise sequence placements^13^. To reduce these adverse effects, 982 species from a wide range of distinct environments were selected to build the reference tree in our study (Supplementary Figure S1). Compared to the method used previously^13^, this strategy expanded the phylogenetic breadth from 23 to 30 phyla, considerably increasing the representation of free-living organisms from the relevant environments and the accuracy of sequence placement. Moreover, in order to ensure the topological reliability, we adopted the strategy of using a convincing starting tree of 250 tips based on the previous study^13^ when building the reference tree. As a result, more than 70% of the branches of our maximum likelihood tree had high bootstrap supports (>80%), and the relative species representative of different phyla could be well separated into clear monophyletic groups (Supplementary Figure S1). As such, the resolution of our reference tree was sufficient to gain reliable results.

Our results have demonstrated that the rERs of naturally occurring communities were habitat-dependent. Although the samples belonging to the same habitat were widely distributed (Supplementary Table S2), their signals of community rER were consistent regardless of the long geographic distance, suggesting the importance of environmental conditions to the evolutionary pattern. Parallel evolution driven by environmental conditions might be a reasonable explanation for this observation. Similar traits have been reported to be parallelly developed in related but distinct species under similar environmental selections^22^. In supporting this, a previous study of microbial laboratory evolution has found a strong pattern of convergence at the level of genome content under the same selective pressure^23^. In the current study, natural communities from a specific habitat presumably suffer from similar selections and these habitat-specific pressures plausibly facilitate the adaptation of microorganisms to the environment. Indeed, increasing evidence has demonstrated that free-living microbes are subject to parallel evolution to respond to environment with high temperature^24^,^25^ and habitat-specific selective environment in *Methylobacterium*^26^ which were widely distributed in soil and freshwater.

Perhaps the most interesting finding of this study is that microbial communities from extreme environments evolve faster than those from normal habitats. While it might be argued that such a conclusion made via the estimation of rERs is doubtable, some previous studies addressing the absolute rates of molecular evolution in extreme environments partly support this result. For example, the *in situ* measured genome-wide substitution rate for *Leptospirillum* bacterium from an AMD community was approximately 1.4 × 10^−9^ per site per generation^9^, which was fairly high for free-living bacteria from natural environments. For previous study revealed that this rate was just a little lower than that of symbiotic and pathogenic associations^27^, which was thought to evolve extremely fast.

Existing studies suggest that genome size scales negatively with mutation rates^28^. Our additional analyses showed that organisms in extreme environments tend to exhibit a trend of smaller average genome size compared to those in normal environment (2.72 Mb vs. 3.13 Mb) (Supplementary Table S3), but this pattern is not significant, presumably due to a specific case of extensive genome “streamlining” in ocean surface communities^29^. Another explanation for the contrasting community rERs between the two habitat groups might be that the organisms inhabiting extreme environments had lower effective population sizes (*Ne*). Although direct measurement of *Ne* was not possible in the current study due to the limited genetic information on the microbial populations between the two groups, whether *Ne* is a determinant for shaping this evolutionary pattern among distinct habitats merits future study. Additionally, some other critical clues found in our study may, to some extent, explain the observation of high community rERs in extreme environments. As it was previously suggested that natural selection is less efficient in small populations^30^, our results supposed that relaxed purifying selection might occur more frequently in extreme environments because of the relatively smaller population sizes. Similarly, a more relaxed selective constraint was found in microbial communities in the deep sea rather than the surface water^19^. Generally, relaxed selective constraint increases the proportion of low frequency variants^31^ that might directly contributes to higher mutation rates, and this effect could facilitate the evolution of phenotypic plasticity^32^. Thus, relaxed purifying selection might be a common evolutionary strategy accelerating the adaptive response of microbes to extreme environments by increasing their metabolic versatility. Another explanation accounting for fast evolution in extreme habitats was the higher frequency of HGTs. Theoretically, higher frequency of gene recombination would directly lead to more extensive variation of gene content (such as the formation of mosaic genomes^10^), and the high genomic divergence of microbes comparing to their relatives in the reference tree would consequently raise the rERs. In this study, the level of transposases that representing the frequency of gene transfers was significantly higher in extreme environments. Furthermore, our odds ratio analysis also suggested the gene enrichment involving in recombination in extremely acidic communities (e.g., AMD, Figure 7). Overall, frequent recombination might be an alternative strategy enabling rapid adaptation of microbes to extreme conditions^9^.

The contrasting community rERs between extreme and normal environments may reflect distinct evolutionary histories as well. As microorganisms populating relatively benign environments have likely reached a steady-state of environmental adaptation, most mutations may have deleterious or neutral effects on fitness and thus have limited opportunities for fixation^33^,^34^. In contrast, the fitness of microbes inhibiting more stressful environments is far from optimal, and thus adaptive evolution is expected to occur more frequently^34^,^35^. For example, AMD environments are typically characterized by extremely low pH and heavy metal toxicity, which are stressful for the growth of microorganisms. Thus, adaptation by merely fixing pre-existing variations could not meet the demand of innovation^36^, resulting in relatively high community rERs. However, the remarkably high frequency of mutations could also lead to a high genetic load because of the accumulation of excessive deleterious mutations^37^. Consequently, to counteract the effects of deleterious mutations, the cost of balancing the necessity of adaptive changes is presumably high. Two evolutionary signals observed in our AMD communities may support this assumption. Firstly, the overrepresentation of genes related to repair systems in existing taxa (Figure 7) might counteract the high rates of stress-induced mutations. Secondly, there is clear evidence suggesting that mismatch repair (MMR) genes lost or inactivated during early colonization may be restored by HGTs^38^, reflecting a compensatory strategy to redress this balance. Collectively, the accelerated evolution implies the ongoing adaptation of microbes living in extreme environments.

Our community-scale evolutionary study across distinct habitats suggested that the evolutionary rate of microbial communities under extreme conditions was higher. This seemed, to some extent, inconsistent with previous reports that (hyper)thermophilic organisms generally exhibited lower mutation rates compared with mesophiles^39^. Indeed, evidences implied a relatively low evolutionary rate of (hyper)thermophiles possibly due to their unusual evolutionary pattern such as distinct mutational spectra^40^,^41^ and repair strategies^42^. In this study, only microbes dwelling in hot spring were thermophiles with an average optimal growth temperature (OGT) > 50°C, while the others in diverse habitats including AMD, saline lake, surface ocean, freshwater and soil were mesophiles (The community average OGT was estimated based on previous methods^7^). Statistically, our additional analysis supported the previous idea when comparing the average evolutionary rate of hot spring communities with that of other habitats (0.117 vs. 0.226, t-test, *P* = 0.0023, α = 0.001, one-tailed). However, this result was habitat-dependent. For example, although the thermophiles in hot spring had a lower evolutionary rate than mesophiles from extreme environments like AMD and saline lake, they significantly evolved faster than mesophiles in normal habitat such as soil (detailed *P* values see Figure 2). Thus, results made by previous classical studies focusing on a single environmental factor (e.g., temperature) may not always be convinced. It should be noted that the evolution of microorganisms in natural environments are shaped by multiple environmental factors, and our comparison of community-scale evolutionary rates between “extreme” and “normal” habitats highlight their integrated impact on microbial evolution. Consequently, our conclusion is still reasonable and from the point of view of this study, thermophiles do not necessarily evolve slower than mesophiles at the community level.

Our current study has provided a significant insight into the evolutionary mechanisms underlying the adaptation of microbes to extreme environments. This promising framework extended from previous approaches highlights the importance of exploring the evolutionary processes of microorganisms at the community level. Meanwhile, we recognized the potential bias associated with using the midpoint rooting method, as considerable variations of evolution rates may exist across the full spectrum of phylogeny. Additionally, the number of phylogenetic marker gene sequences detected from the metagenomic samples was relatively small. Thus, the observed patterns may be largely contributed by the dominant taxa and thus could not comprehensively reflect the overall community structure particularly for the complex habitats. Finally, some important population genetic parameters such as effective population size and generation time were not assessed in this study because of the technical limitations of their precise estimation at the community level. In sum, the application of our framework addressing the evolution processes of the overall community is feasible to reveal the evolutionary mechanisms of natural microbial communities. Future studies may benefit from the quantitative evaluation of evolutionary life history traits and more exhaustive sampling of genetic content using high throughput sequencing.

## Methods

### Dataset acquisition and metagenomic analyses

The metagenomic sequences from 40 samples across six habitats were downloaded from the NCBI SRA, MG-RAST, IMG/M and CAMERA databases (Supplementary Table S2). Only prokaryotic sequences were retrieved for the subsequent analyses. All these sequences were generated by 454 platform except for those from six samples which were generated by Sanger sequencing. For the 454 pyrosequencing data, raw reads were trimmed with an average Phred quality score < 20, and quality sequences were de-replicated using a 454 replicate filter^43^. The sequence assembly was carried out using the Newbler de novo assembler (version 2.6) with default parameters. The resulting contigs and singletons ≥ 300 bp and all the Sanger sequences were further analyzed as described below: (1) For taxonomic binning, sequences were compared against the NCBI-nr database using BLASTX, then the species diversity estimated by ACE index was calculated following the pipeline of QIIME^44^. (2) For functional annotation, the protein encoding genes were firstly predicted using GeneMark^45^. The predicted protein sequences were then compared against the STRING (Version 9.0)^46^ database using BLASTP with a reliable hit standard as “match length ≥ 100, identity ≥ 50%, coverage ≥ 50% and BLAST score ≥ 60”. The hits were assigned to the corresponding Clusters of Orthologous Group (COG) catalogues and COG categories.

### Average genome size (AGS) estimation

The average genome size for each metagenomic sample was estimated as previously described^47^. Firstly, reads sequences were directly BLASTX against STRING database, and the number of hits annotated as phylogenetic marker was counted. Then the average genome size was calculated based on the equation as below: 
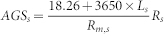


Where *L_s_* denotes the average read length of sample *s*, *R_m,s_* stands for the number of reads annotated as phylogenetic marker *m* from sample *s*, and *R_s_* represents the total number of base pairs sequenced from sample *s*.

### Functional clustering of microbial communities across different habitats

To assess the functional distribution pattern of microbial communities across different habitats, functional clustering was performed using a discriminant analysis of principal components (DAPC) in R package “adegenet”^48^ based on the relative abundances of the COG categories.

### Detection of phylogenetic marker genes

A set of 31 well-defined phylogenetic marker genes described previously by Ciccarelli *et al*.^49^ was suggested as the estimator of rER in natural communities^13^. In this study, we scanned these phylogenetic markers for the subsequent community rER measurement based on the annotated COG catalogue information. Generally, 952 ± 2363 (mean ± sd) phylogenetic marker sequences were detected among the 40 samples.

### Reference species selection

To establish a robust reference phylogeny for assessing the community rER, 982 species including 883 bacteria, 69 archaea and 30 eukaryotes from the STRING (version 9.0)^46^ database were selected to build the reference tree. The 31 phylogenetic marker sequences were retrieved from these species, of which none were reported to have potentially undergone HGTs in these phylogenetic markers^50^. The 982 species were sampled from a wide range of distinct environments and cover the most major 30 prokaryotic phyla, thus considerably increasing the representation of free-living organisms from the relevant environments.

### Building concatenated phylogenetic marker alignment

Based on the approach described by Ciccarelli *et al*.^49^, the alignments were built respectively for the 31 phylogenetic maker sequences from the 982 genomes using muscle^51^ and then concatenated. Gaps and poorly aligned regions were eliminated using Gblocks^52^ with the same parameters described by Ciccarelli *et al*.^49^, and finally 5475 positions were remained in the alignment.

### Reference tree construction and sequence placement

A maximum likelihood tree was constructed based on the concatenated alignment mentioned above using Raxml^53^ (version 7.2.7) with the evolution model WAG + G8 + Invariable + F. The topological consensus was assessed using 100 bootstrap replicates on a parallel cluster. A well-established starting tree (250 tips) that covers major microbial phyla from the previous study^13^ was used as a priori method to improve the topological accuracy during the reference tree construction. The root was determined by the method of automatic mid-point rooting using the R package of “phangorn”^54^. The branch length was calculated using “adephylo” package^55^ in R. Before placement, each individual sequence of the 31 phylogenetic markers detected from the 40 samples was re-aligned respectively based on the concatenated alignment using hmmalign^13^. Then according to the new alignment, this sequence could be placed onto the reference tree using pplacer^56^.

### Quantitative phylogenetic assessment of community rERs

Branch length indicates the accumulated number of sequence changes in a rooted tree. Based on the approach previously reported by von Mering *et al*.^13^, the rER of each phylogenetic marker was inferred from the branch length variations between the query sequence and the median of those of all relatives in the same phylum from the reference tree. Accordingly, the community rER of each sample could be assessed as the median of the rERs of all phylogenetic markers, while the mean of the community rERs of samples from the same habitat reflected the overall rER of that specific habitat type (see detailed pipeline for community rER estimation in Supplementary Figure S2).

### Simulation analysis of community rER

To test the influence of community composition on community rER, all phylogenetic marker sequences from a specific habitat were pooled and randomly assigned to each relevant community according to its phylogenetic composition^57^. The number of sequences that was used to re-create the communities was based on the smallest dataset among the samples from the same habitat. The expected community rERs of the simulated communities were estimated and compared to the observed values using two-sided Kolmogorov-Smirnov tests.

### Detection of natural selection signature

For each community, all orthologous proteins were aligned using muscle^51^, and the ratio of nonsynonymous to synonymous substitutions between orthologs (dN/dS) was calculated using PAML^58^, which was used to infer the natural selection force.

### Assessment of community-wide HGTs

Previous approaches based on substitution distribution or phylogeny for inferring the HGT events largely addressed certain genes or taxa using whole genome data with scaffolds larger than 10 kb^59^,^60^. No relevant studies have characterized the overall HGT at the community level with short reads (typically less than 1 kb) derived from metagenomic sequencing. In this study, the transposase level, which was previously suggested to correlate with the frequency of HGT^8^,^15^,^20^, was used as an approximate proxy for the assessment of community-wide HGTs. Prior to the transposases level calculation^20^, we tested the correlation between the number of transposases and the HGT events of 328 complete prokaryotic genomes based on the dataset retrieved from a previous study^50^. The positive relationship (see Supplementary Figure S3) implied that the transposases level could be used as an alternative estimator to assess the community-wide HGTs.

### Clustering analysis

An exploratory clustering analysis of all the 40 samples was conducted using R package “hclust” according to the four evolutionary indexes including community rERs, dN/dS, HGTs and species diversity (Supplementary Table S1). The result was visualized with FigTree (http://tree.bio.ed.ac.uk/software/figtree/).

### AMD community evolutionary analysis

The AMD habitat was selected to perform detailed analyses to reveal the potential link between evolutionary adaptation and environmental conditions at the community level. The odds ratio method described by Hemme CL *et al*.^8^ was used to detect the genes enriched in the AMD habitat by comparing genes that were assigned to COG functional categories from all 10 AMD communities against those from all the sequenced prokaryotes genomes in IMG. The result was visualized as ln (odds ratio) with positive and negative trends denoting over- and under-representation, respectively. The significance was assessed using one-tailed Fisher's exact test.

### Average optimal growth temperature (OGT) estimation

The community average OGT for each metagenomic sample was estimated based on previous methods^7^ as follow: OGT = 937F-335, where F denotes the average fraction of amino acids sets (IVYWREL) in the total protein sequences of each metagenome.

## Author Contributions

S.J.L., Z.S.H. and W.S.S. conceived the study; Z.S.H. and S.J.L. performed the analysis; L.N.H., J.Li, L.X.C., J.Liu, K.J.L., M.H. and S.H.S. assisted with the data analysis; and S.J.L. and W.S.S. wrote the manuscript.

## Supplementary Material

Supplementary Information Supporting Information
