# Supplementary material for: Microbial communities evolve faster in extreme environments
Source: Sci Rep. 2014 Aug 27;4:6205. doi: 10.1038/srep06205 (PMC4145313; doi:10.1038/srep06205)
Supplement: Supplementary Information — Supporting Information [file srep06205-s1.pdf]

Supporting Information for

Microbial communities evolve faster in extreme environments

Sheng-Jin Li[†], Zheng-Shuang Hua[†], Li-Nan Huang, Jie Li, Su-Hua Shi, Lin-Xing Chen, Jia-Liang Kuang, Jun Liu, Min Hu & Wen-Sheng Shu[*]

State Key Laboratory of Biocontrol, Key Laboratory of Biodiversity Dynamics and Conservation of Guangdong Higher Education Institutes, College of Ecology and Evolution, Sun Yat-sen University, Guangzhou 510275, People's Republic of China

[†]These authors contributed equally to this work.

[*]Corresponding author: Wen-sheng Shu (W.S.S.), College of Ecology and Evolution, Sun Yat-sen University, Guangzhou 510275, PR China.

Tel.: +86 20 39332933;

Fax: +86 20 39332944;

E-mail: shuws@mail.sysu.edu.cn

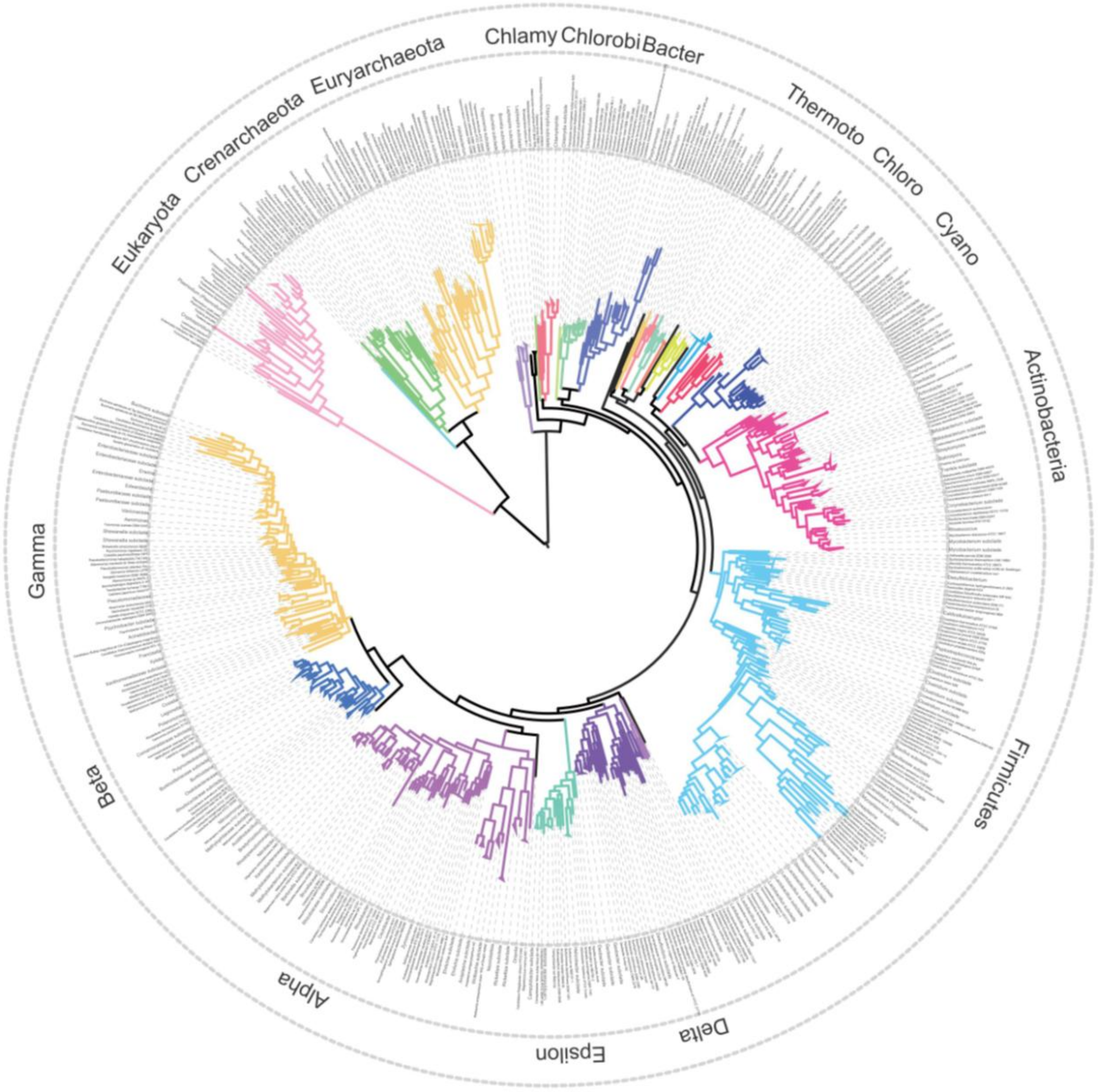

Supplementary Figure S1 | A maximum likelihood genome tree of 982 species (including 883 *Bacteria*, 69 *Archaea and* 30 *Eukaryote*) used as reference tree for computing rERs. The tree was constructed from the concatenated protein sequence alignments derived from 31 marker genes of the genomes. Each phylum comprised a separate monophyletic group and different groups were distinguished using different colors.

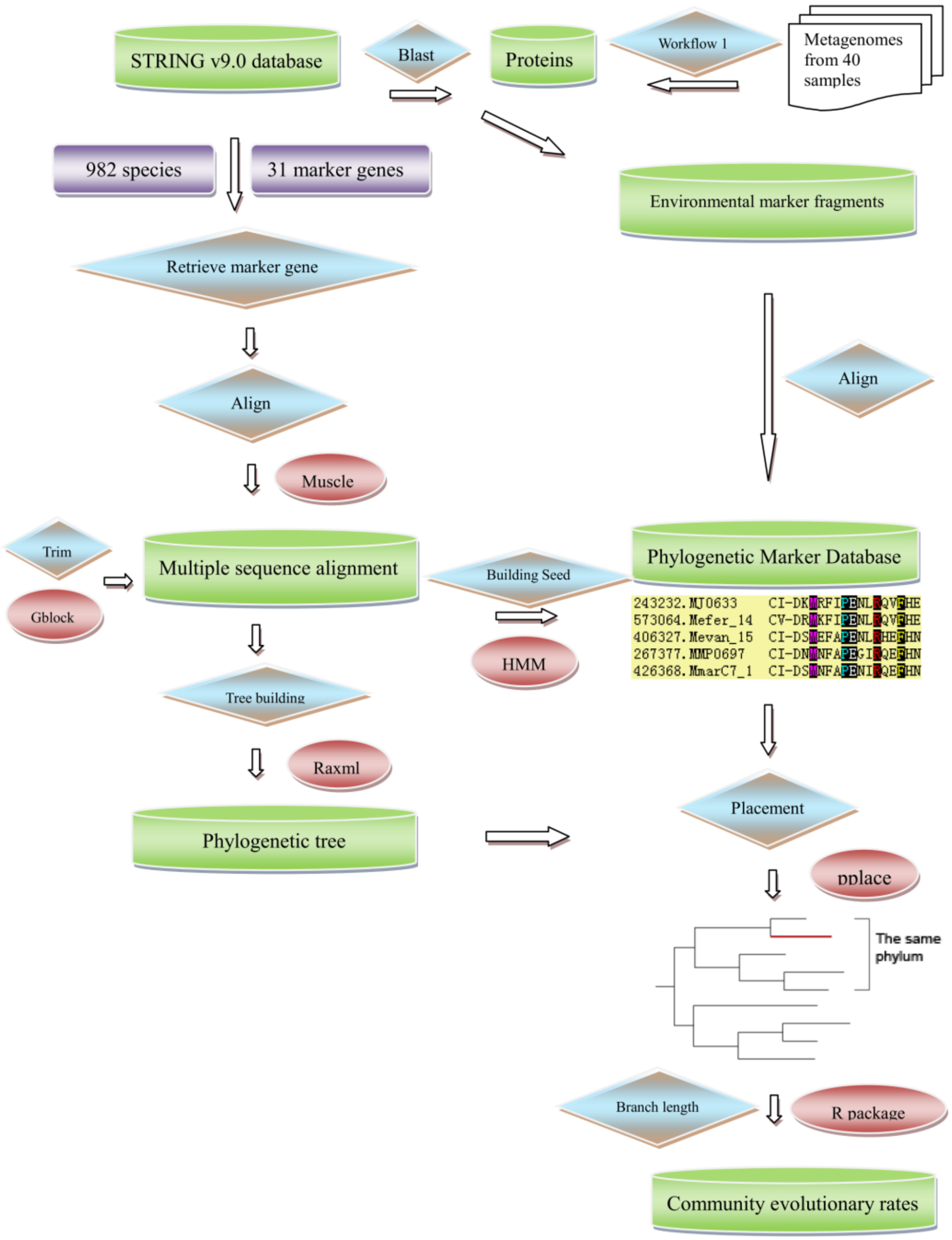

Supplementary Figure S2 | Detailed pipeline for community rERs estimation. The environmental marker fragments from 40 metagenomes were placed onto a custom concatenated reference tree of 982 species using pplacer[1]. The community rERs were estimated by comparing the branch length of the environmental fragment with their relatives within the same phyla on the reference tree based on a previous study[2].

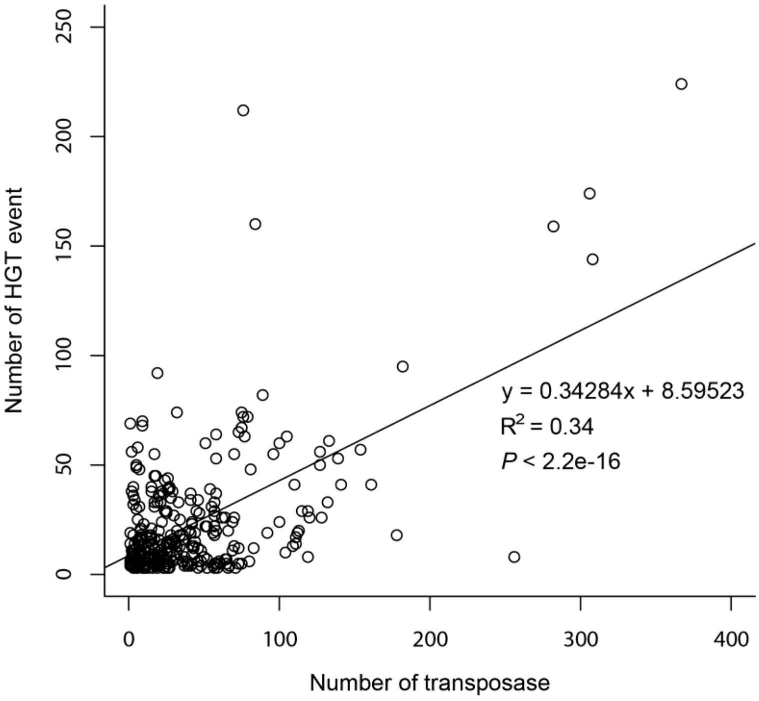

Supplementary Figure S3 | Positive correlation between the number of transposases and HGT events observed in 328 prokaryotic microbes. The data on HGT events in these prokaryotic genomes was retrieved from a previous study[3]. For each genome, the number of transposases was calculated based on BLAST all genes against a database of transposase protein families with an expectation value cut-off of 10^{-5} as previous described[4]. The correlation analysis was used as a support for approximate assessment of community-wide HGTs based on the transposases level in metagenome.

Supplementary Table S1 | Detailed community-scale evolutionary data of community rERs, dN/dS, transposase level and species diversity for all 40 samples.

Habitat	Sample	rER	Transposases (%)	dN/dS	Species diversity (ACE)
Saline Lake	GSL NARP	0.540	0.917	0.122	54
	Saltern SS37	0.520	0.479	0.132	91
	GSL NAS	0.453	0.782	0.071	94
	Saltern SS19	0.263	0.439	0.068	141
	GSL SAAI	0.211	0.551	0.139	130
	GSL SAS	0.105	0.857	0.078	198
	Average ±sd	0.349 ±0.180	0.671 ±0.206	0.102 ±0.033	118 ±50
AMD	C75 0806	0.427	0.577	0.123	170
	C75 0807	0.417	0.916	0.080	155
	C75 0507	0.356	1.219	0.114	234
	C75 0606	0.363	0.921	0.129	79
	C75 1106	0.337	1.123	0.100	193
	FK	0.395	0.443	0.217	119
	YFP	0.359	0.751	0.201	224
	5way	0.275	2.320	0.156	87
	DBS	0.223	0.839	0.188	217
	YFS	0.214	0.911	0.178	234
	Average ±sd	0.337 ±0.076	1.002 ±0.517	0.149 ±0.046	171 ±59
Hot spring	Bison Site 1	0.122	0.873	0.148	163
	Bison Site 3	0.109	0.777	0.117	139
	Bison Site 4	0.170	0.434	0.113	135
	Bison Site 5	0.067	0.959	0.127	183
	Average ±sd	0.117 ±0.042	0.761 ±0.230	0.126 ±0.015	155 ±22
Surface ocean	BATS 100m	0.233	0.021	0.058	221
	BATS 20m	0.234	0.039	0.035	247
	BATS 50m	0.222	0.027	0.066	262
	HOT 110m	0.242	0.020	0.060	288
	HOT 25m	0.228	0.032	0.085	287
	HOT 500m	0.242	0.053	0.089	338
	HOT 75m	0.241	0.025	0.072	267
	Sargasso Sea	0.144	0.252	0.043	237
	Monterey Bay	0.141	0.066	0.038	119
	Average ±sd	0.214 ±0.041	0.059 ±0.074	0.061 ±0.020	252 ±60
Freshwater	Yellowstone Lake	0.125	0.027	0.072	162
	Amazon River	0.095	0.210	0.060	108
	Lake Lanier	0.085	0.263	0.072	177
	UlvaLactuca	0.097	0.195	0.143	238
	Average ±sd	0.100 ±0.017	0.174 ±0.102	0.087 ±0.038	171 ±53
Soil	Luquillo Forest	0.060	0.900	0.105	231
	F1	0.081	0.727	0.099	231
	J1	0.041	0.727	0.100	304
	J1a-10	0.015	0.687	0.097	293
	J1b-10	0.074	0.753	0.086	256
	Harvard Forest	0.032	0.766	0.070	227
	Waseca Soil	0.032	0.614	0.050	317
	Average ±sd	0.048 ±0.024	0.739 ±0.087	0.087 ±0.020	265 ±38

The average number and standard deviation (sd) of each parameter for the six habitat categories are also shown.

Supplementary Table S2 | Metagenomic dataset of the 40 samples used in this study.

Environment	Sample ID	Accession	Sample Site	Sequencing Platform	Database
AMD	5way AMD	4441137.3	Richmond mine, California, USA	Sanger	MG-RAST
	C75 0507	SRP009106		454 GS FLX Titanium	SRA NCBI
	C75 0606				
	C75 0806				
	C75 0807				
	C75 1106				
	DBS	SRS212548	Guangdong, China	454 GS FLX	
	FK	SRS212549			
	YFS	SRS212550			
	YFP	SRS212551			
Saltern Lake	Saltern SS19	SRP007685	Santa Pola Solar Saltern	454 GS FLX Titanium	
	Saltern SS37				
	GSL NARP	IMG Submission ID:1452	NARP,Utah	454 Titanium,short-insert Sanger	IMG
	GSL NAS				
	GSL SAS				
	GSL SAAI				
Hot Spring	Bison Site 1	CAM_PROJ_BisonMetagenome	Yellowstone National Park, USA	Sanger	CAMERA
	Bison Site 3				
	Bison Site 4				
	Bison Site 5				
Ocean	BATS 100m	SRP001096	BATS216	454 GS FLX Titanium	SRA NCBI
	BATS 20m				
	BATS 50m				
	HOT 110m	SRP001041	HOT186		
	HOT 25m				
	HOT 500m				
	HOT 75m				
	SargassoSea	AACY01000001–AACY01811372	Sargasso sea	454 GS FLX	SRA NCBI
	Monterey Bay	Monterey Bay Microbial Study	Monterey Bay		MG-RAST
Freshwater	Amazon River	SRP005263,SRR091234	Amazon river	454 GS FLX Titanium	SRA NCBI
	Lake Lanier	SRP003195:SRR063691	lake Lanier, Atlanta		
	UlvaLactuca	SRP003006	Ulva lactuca, Australia		
	Yellowstone Lake	SRP004789	Yellowstone, USA		
Farm Soil	Waseca Soil	CAM_PROJ_FarmSoil	Waseca County, Minnesota, USA	Sanger	
Forest Soil	Luquillo Forest	SRR034258	Luquillo Forest	454 GS FLX Titanium	
	Harvard Forest	sra028811	Harvard Forest，USA	454 GS FLX	
Grassland Soil	F1	download address*	Hertfordshire, UK	454 GS FLX Titanium	
	J1				
	J1a-10				
	J1b-10				

* http://www.genomenviron.org/Projects/METASOIL.html

Supplementary Table S3 | Average genome size for all 40 metagenomic samples.

Environments (Mean ± sd)	Habitats (Mean ± sd)	Sample	Average genome size (Mb)
Extreme (2.72 ± 0.79)	Saline lake (2.27 ± 0.24)	GSL NARP	1.95
		Saltern SS37	2.58
		GSL NAS	2.40
		Saltern SS19	2.02
		GSL SAAI	2.34
		GSL SAS	2.31
	AMD (2.57 ± 0.67)	C75 0806	4.00
		C75 0807	2.18
		C75 0507	2.97
		C75 0606	2.69
		C75 1106	2.27
		FK	2.33
		YFP	2.28
		5way	3.26
		DBS	1.72
		YFS	2.05
	Hot spring (3.78 ± 0.74)	Bison Site 1	3.81
		Bison Site 3	3.75
		Bison Site 4	2.86
		Bison Site 5	4.67
Normal (3.13 ± 1.73)	Surface ocean (1.56 ± 0.21)	BATS 100m	1.44
		BATS 20m	1.24
		BATS 50m	1.46
		HOT 110m	1.77
		HOT 25m	1.47
		HOT 500m	1.73
		HOT 75m	1.46
		Sargasso Sea	1.56
		Monterey Bay	1.91
	Freshwater (3.06 ± 1.1)	Yellowstone Lake	2.60
		Amazon River	2.23
		Lake Lanier	2.75
		UlvaLactuca	4.68
	Soil (5.19 ± 0.45)	Luquillo Forest	6.15
		F1	4.97
		J1	5.13
		J1a-10	4.98
		J1b-10	4.77
		Harvard Forest	5.15
		Waseca Soil	5.19

The average number and standard deviation (sd) for the six habitat categories are also shown.

References:

1. Matsen, F. A., Kodner, R. B. & Armbrust, E. V. pplacer: linear time maximum-likelihood and Bayesian phylogenetic placement of sequences onto a fixed reference tree. *BMC Bioinformatics* **11,** 538 (2010).
2. von Mering, C. *et al*. Quantitative phylogenetic assessment of microbial communities in diverse environments. *Science* **315,** 1126-1130 (2007).
3. Smillie, C. S. et al. Ecology drives a global network of gene exchange connecting the human microbiome. *Nature* **480**, 241-244 (2011).
4. Brazelton, W. J. & Baross, J. A. Abundant transposases encoded by the metagenome of a hydrothermal chimney biofilm. *ISME J.* **3,** 1420-1424 (2009).
